# Supplementary material for: Dendritic atoh1a+ cells serve as Merkel cell precursors during skin development and regeneration
Source: Development. 2025 Jun 20;152(20):dev204810. doi: 10.1242/dev.204810 (PMC12212647; doi:10.1242/dev.204810)
Supplement: Supplementary information [file develop-152-204810-s1.pdf]

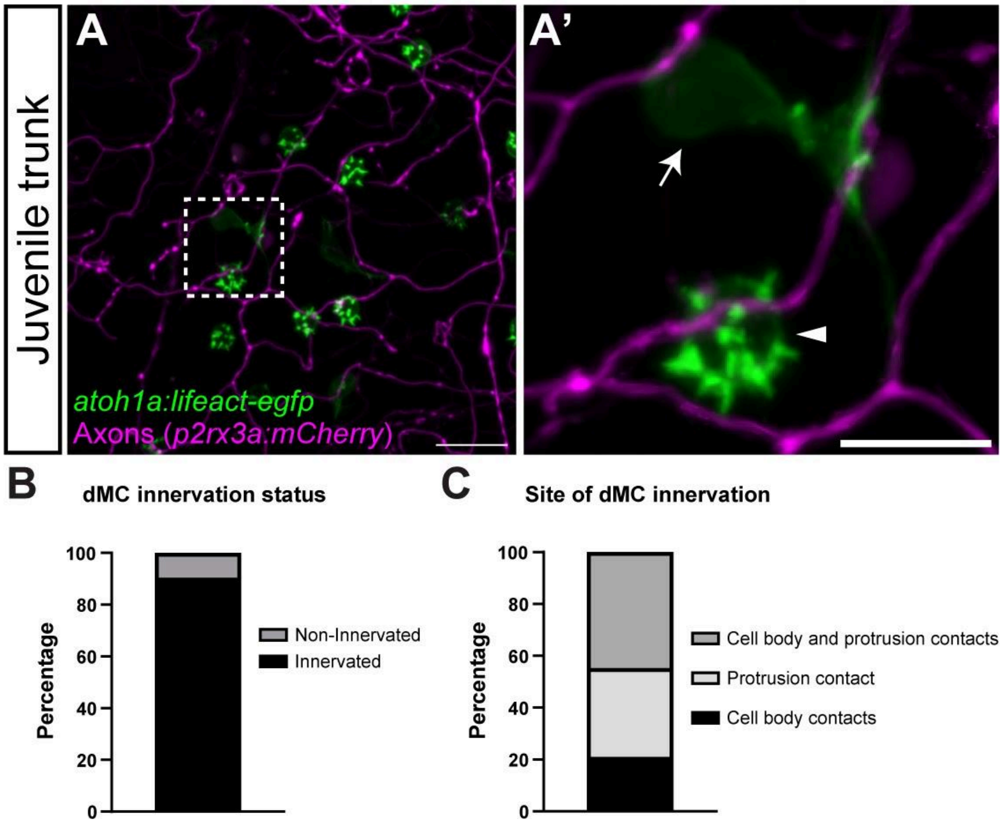

Fig. S1. Somatosensory innervation of dMCs.

(A) Representative images of *atoh1a*+ cells (green; *Tg(atoh1a:lifeact-egfp)*) and somatosensory axons (magenta; *Tg(p2rx3a:mCherry)*) along the lateral trunk at 16 mm SL. White dashed box indicates the region magnified in (A'). (A') Magnification shows an innervated MC (arrowhead) and a dMC (arrow) contacting axons both at the cell body and with a protrusion. **(B)** Quantification of percentage of dMCs innervated by *p2rx3a*+ axons (*n*=42 cells from 4 fish; 14-16 mm SL). **(C)** Quantification of site of dMC contact with *p2rx3a*+ axons (*n*=38 cells from 4 fish; 14-16 mm SL). Scale bars, 10 µm (A) and 5 µm (A').

Development • Supplementary information

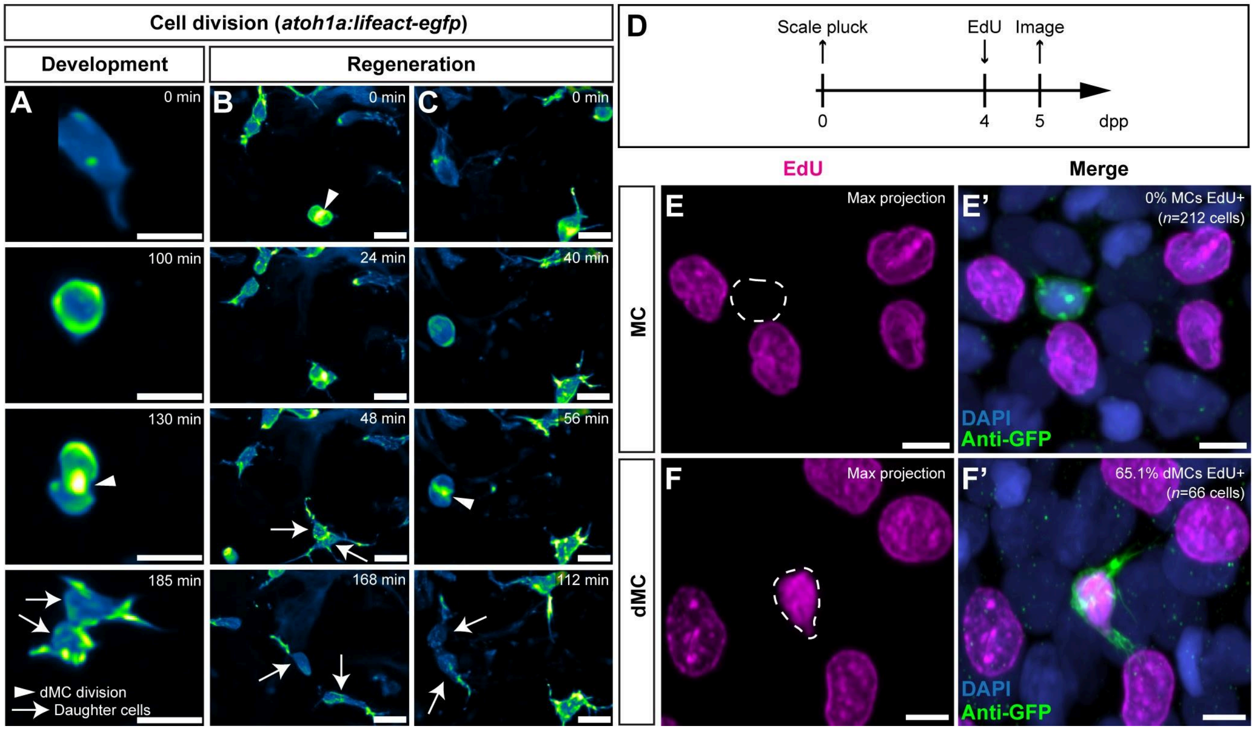

Fig. S2. Differences in cell cycle state of dMCs and MCs.

(A-C) Time lapse stills showing cell divisions of dMCs expressing *Tg(atoh1a:lifeact-egfp)* in juvenile (10.9 mm SL) (A) or regenerating (3 dpp) (B,C) scale epidermis. Arrowheads denote cytokinetic furrows and arrows denote daughter cells. See also Movie 6. **(D)** Experimental design of EdU administration and imaging during scale regeneration. **(E,F)** Representative images of EdU and anti-GFP staining in regenerating *Tg(atoh1a:lifeact-egfp)* scales at 5 dpp. White dashed lines indicate nuclear outlines. *n*=212 MCs and 66 dMCs from 4 fish. Scale bars, 10 µm (A-C) and 5 µm (E-F).

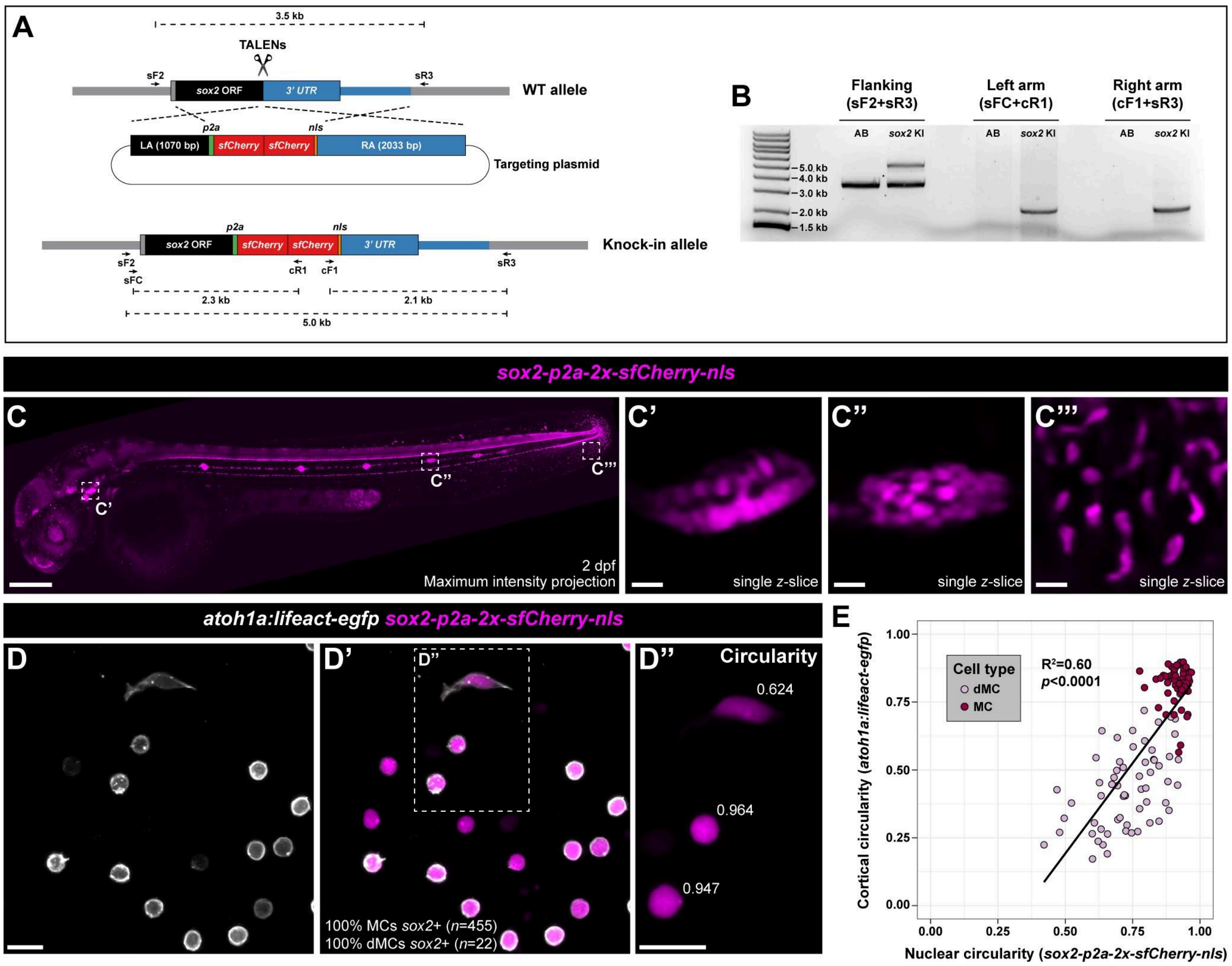

Fig. S3. Generation and analysis of a *sox2* nuclear reporter.

(A) Schematic of the *sox2* locus and TALEN-based strategy to generate the *(sox2-p2a-2x-sfCherry-nls)^{stl1034}* knock-in allele. **(B)** PCR genotyping analysis of genomic DNA lysates from AB and heterozygous *(sox2-p2a-2x-sfCherry-nls)* animals. Primer locations and expected band sizes shown in (A). **(C)** Representative image of *(sox2-p2a-2x-sfCherry-nls)* expression at 2 dpf. Anterior to the left. Dashed boxes indicate regions of magnification: inner ear (C'), posterior lateral line neuromast (C''), fin mesenchyme (C'''). **(D)** Image of the scale epidermis from an adult expressing *Tg(atoh1a:lifeact-egfp)* and *(sox2-p2a-2x-sfCherry-nls)*. All *atoh1a*+ cells also expressed *(sox2-p2a-2x-sfCherry-nls)* in homeostatic skin. *n*=455 MCs and 22 dMCs from 2 fish. Annotations in (D'') represent nuclear circularity values. **(E)** Scatter plot of the correlation between nuclear circularity values measured from *(sox2-p2a-2x-sfCherry-nls)* signal relative to cortical circularity values measured from *Tg(atoh1a:lifeact-egfp)* signal at 5 dpp. Each dot represents a cell (*n*=59 MCs and 61 dMCs from 4 fish), colored according to whether the cell had a dMC or MC morphology. Scale bars, 200 μm (C), 10 μm (C'-C''',D-D'').

Development • Supplementary information

Table S1. Summary of dMC and MC events recorded from live imaging data.

Available for download at
https://journals.biologists.com/dev/article-lookup/doi/10.1242/dev.204810#supplementary-data

Table S2. Linear mixed-effects modeling results related to Fig. 8.

Available for download at
https://journals.biologists.com/dev/article-lookup/doi/10.1242/dev.204810#supplementary-data

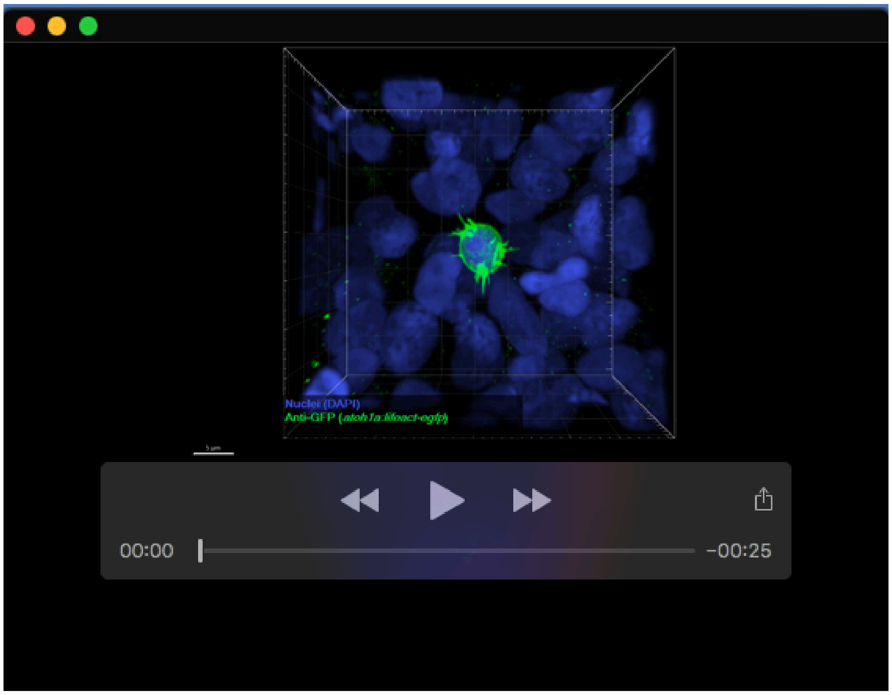

Movie 1. Reconstructed 3D morphology of a zebrafish MC. Representative three-dimensional rotation from a confocal z-stack illustrating MC position and morphology from a scale stained with anti-GFP to label *Tg(atoh1a:lifeact-egfp)* and DAPI to label epidermal nuclei. Arrowheads indicate basal facing microvilli. Scale bar, 5 μm.

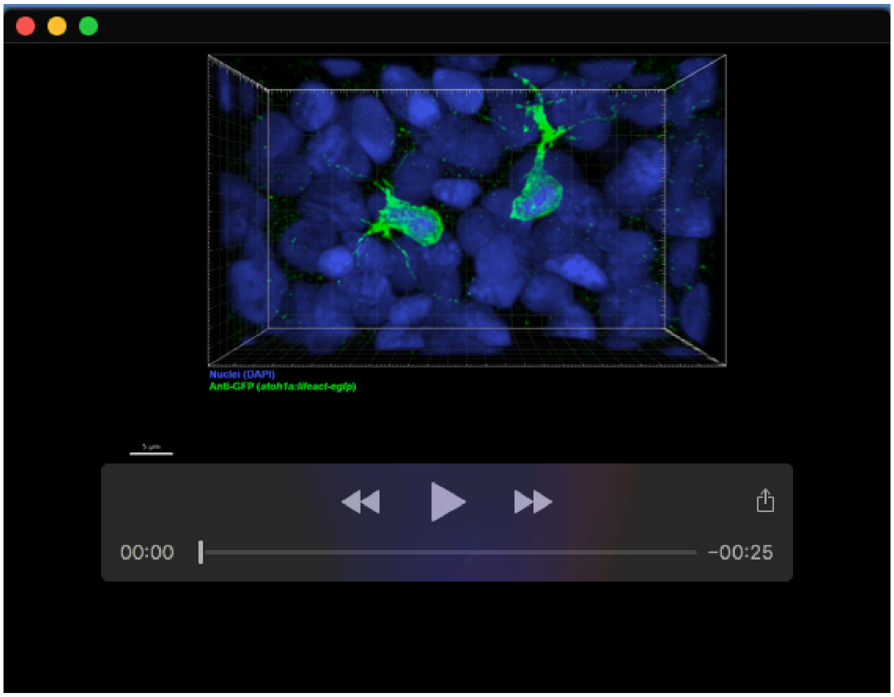

Movie 2. Reconstructed 3D morphology of zebrafish dMCs. Representative three-dimensional rotation from a confocal z-stack illustrating the position and morphology of two dMCs from a scale stained with anti-GFP to label *Tg(atoh1a:lifeact-egfp)* and DAPI to label epidermal nuclei. Arrows indicate laterally and vertically directed protrusions. Scale bar, 5 μm.

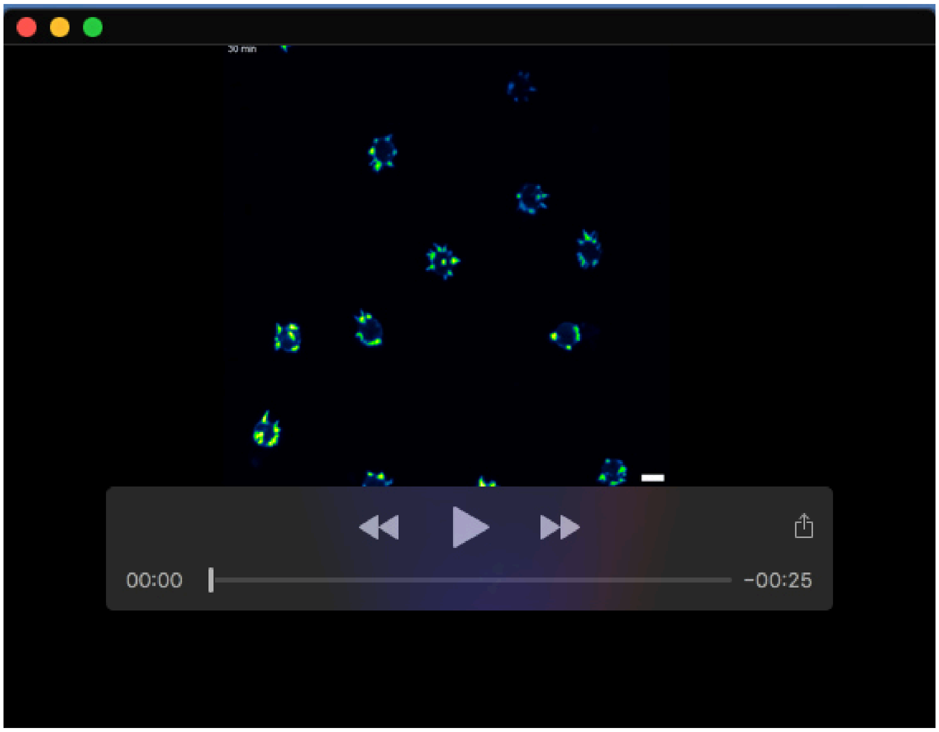

Movie 3. Microvilli dynamics in zebrafish MCs. Time-lapse movie of MCs expressing *Tg(atoh1a:lifeact-egfp)* in juvenile zebrafish skin. Note spike-like microvilli that extend, retract, and coalesce. Scale bar, 5 μm.

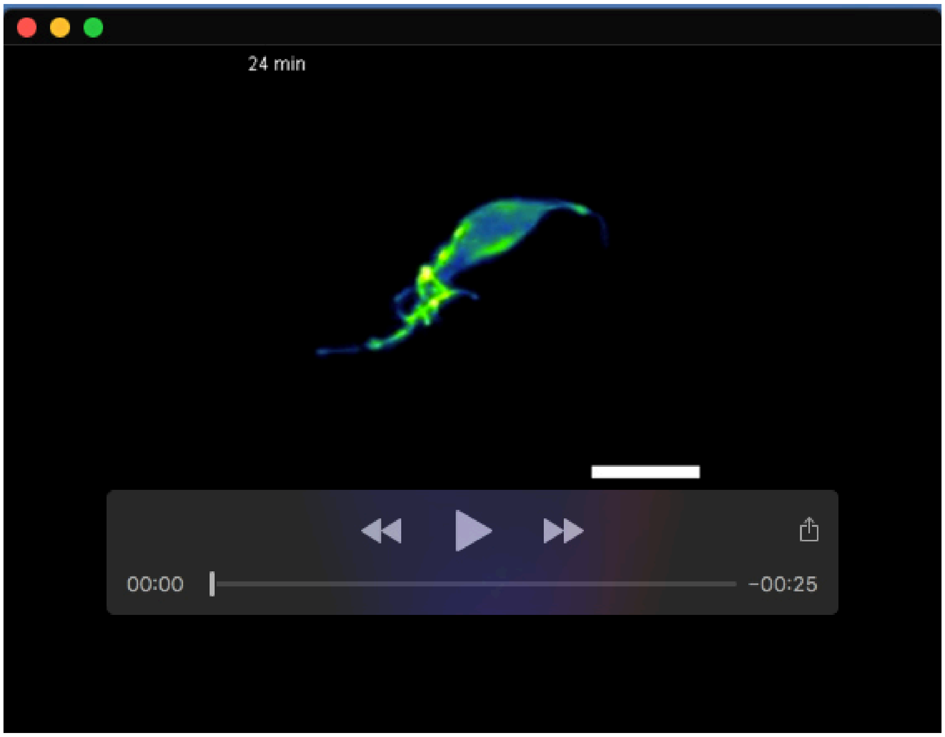

Movie 4. Actin dynamics in zebrafish dMCs. Time-lapse movie of a dMC expressing *Tg(atoh1a:lifeact-egfp)* in juvenile zebrafish skin. Note thread-like filopodial protrusions that are highly dynamic. Scale bar, 10 μm.

Development • Supplementary information

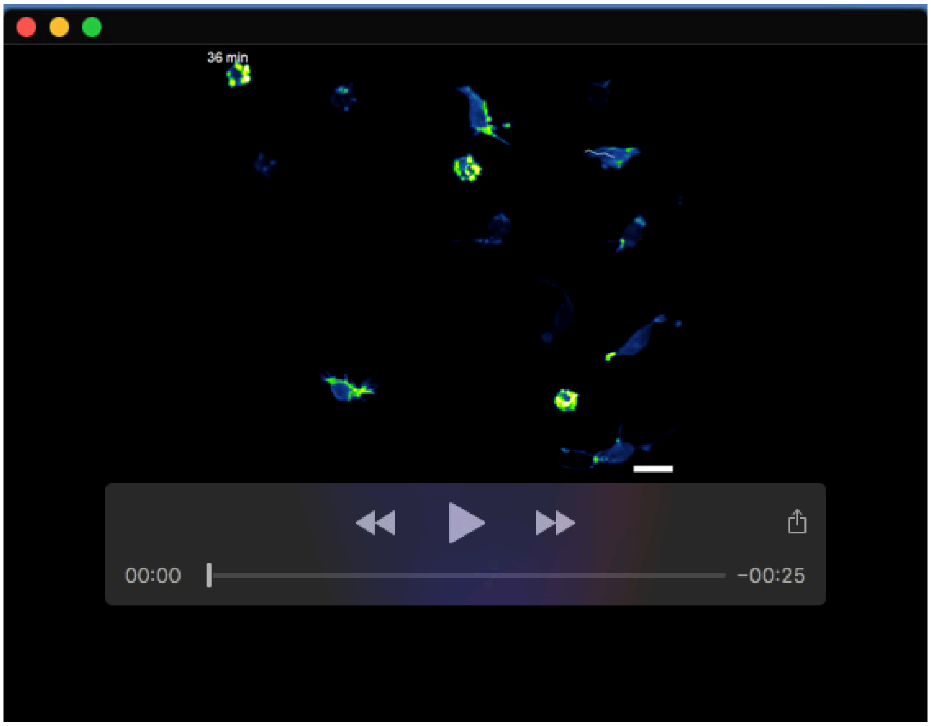

Movie 5. Long-term imaging reveals dMC motile behaviors. 6 hour time-lapse movie of MCs and dMCs expressing *Tg(atoh1a:lifeact-egfp)* in an intubated juvenile zebrafish. Manual cell tracks (white traces) show dMCs crawling in the direction of their protrusions while MCs remain largely stationary. Scale bar, 10 μm.

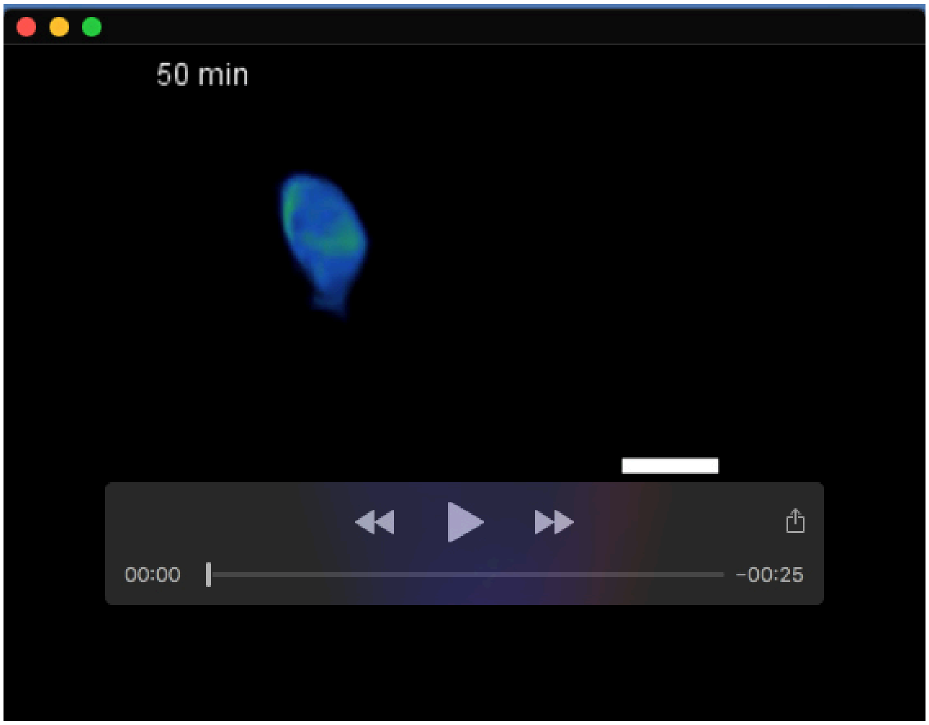

Movie 6. *In vivo* dMC cell division. Time-lapse movie of a dMC expressing *Tg(atoh1a:lifeact-egfp)* undergoing cell division in juvenile zebrafish skin. In all dMC division events recorded, dividing dMCs retract a single unipolar protrusion, form a circular and actin-rich membrane, and undergo cytokinesis. Daughter cells then rapidly extend filopodial protrusions and migrate opposite from one another. Scale bar, 10 μm.

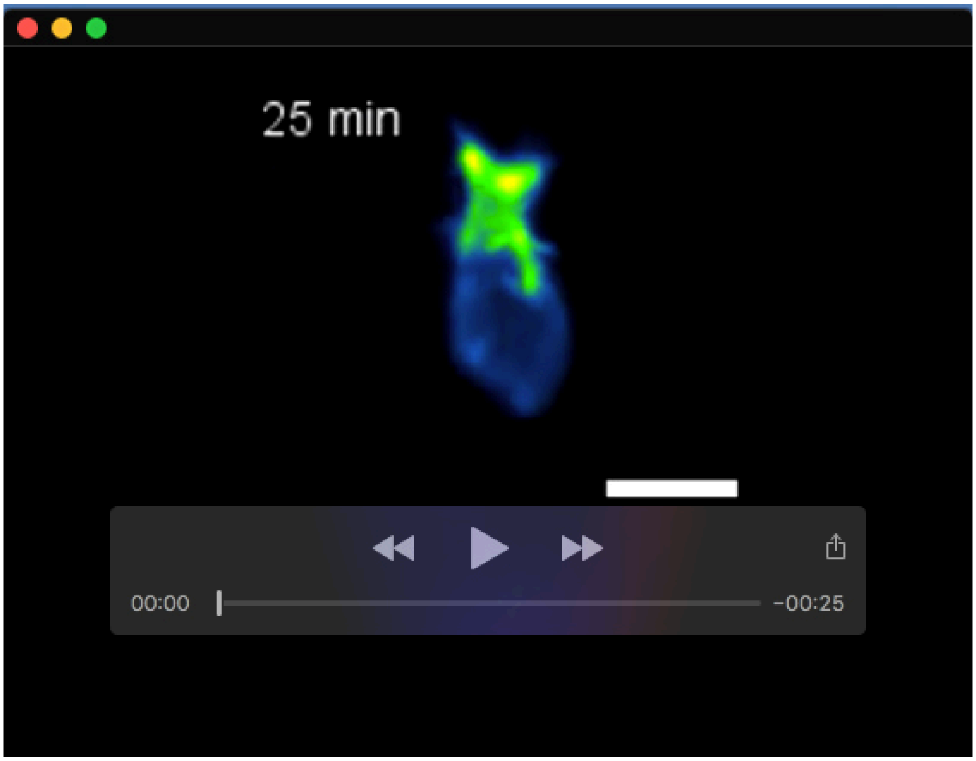

Movie 7. dMC to MC maturation event. Time-lapse of single dMC expressing *Tg(atoh1a:lifeact-egfp)* during scale regeneration that withdraws its filopodial protrusions, rounds its cell cortex, and extends small microvillar extensions as observed in mature MCs. Scale bar, 5 µm.

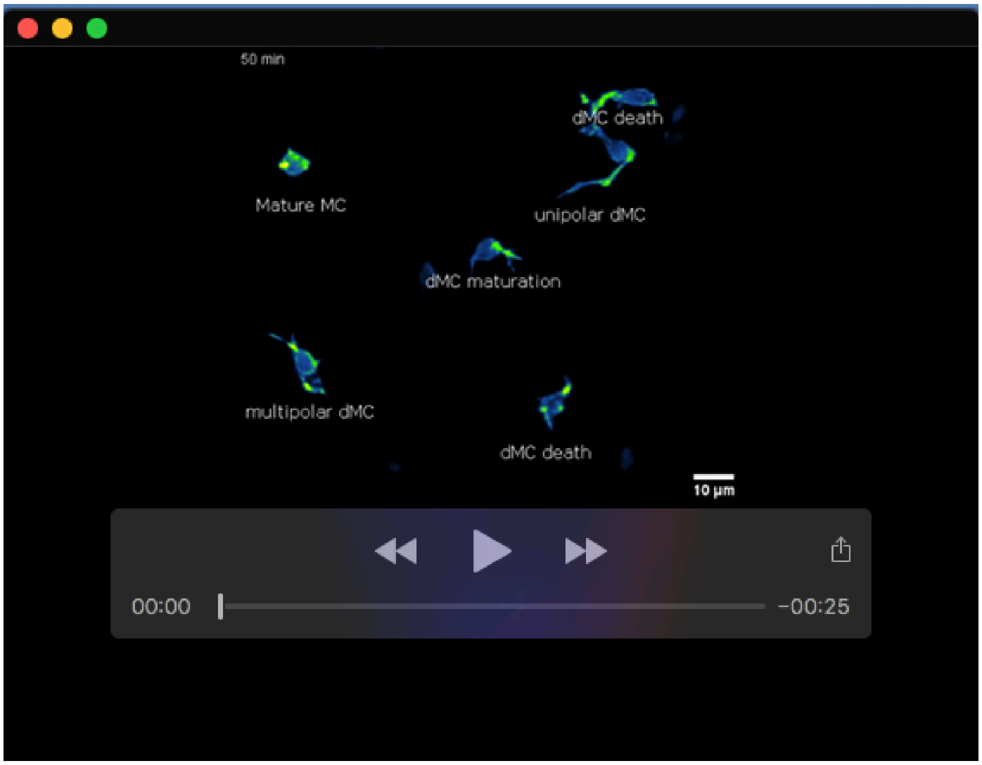

Movie 8. dMC behaviors during regeneration. Time lapse of the regenerating scale epidermis at 3 dpp in a *Tg(atoh1a:lifeact-egfp)* zebrafish. Labels indicate different dMC behaviors including maturation, motility, and cell death. Scale bar, 10 µm.
